# Potential Role of Carotenoids as Antioxidants in Human Health and Disease

**DOI:** 10.3390/nu6020466

**Published:** 2014-01-27

**Authors:** Joanna Fiedor, Květoslava Burda

**Affiliations:** Department of Medical Physics and Biophysics, Faculty of Physics and Applied Computer Science, AGH-University of Science and Technology, al. A. Mickiewicza 30, Kraków 30-059, Poland; E-Mail: kvetoslava.burda@fis.agh.edu.pl

**Keywords:** antioxidant, cancer, cardiovascular disease, β-carotene, carotenoid, chronic disease, oxidative stress, photosensitive disorders, reactive oxygen species

## Abstract

Carotenoids constitute a ubiquitous group of isoprenoid pigments. They are very efficient physical quenchers of singlet oxygen and scavengers of other reactive oxygen species. Carotenoids can also act as chemical quenchers undergoing irreversible oxygenation. The molecular mechanisms underlying these reactions are still not fully understood, especially in the context of the anti- and pro-oxidant activity of carotenoids, which, although not synthesized by humans and animals, are also present in their blood and tissues, contributing to a number of biochemical processes. The antioxidant potential of carotenoids is of particular significance to human health, due to the fact that losing antioxidant-reactive oxygen species balance results in “oxidative stress”, a critical factor of the pathogenic processes of various chronic disorders. Data coming from epidemiological studies and clinical trials strongly support the observation that adequate carotenoid supplementation may significantly reduce the risk of several disorders mediated by reactive oxygen species. Here, we would like to highlight the beneficial (protective) effects of dietary carotenoid intake in exemplary widespread modern civilization diseases, *i.e.*, cancer, cardiovascular or photosensitivity disorders, in the context of carotenoids’ unique antioxidative properties.

## 1. Introduction

Carotenoids (Crts) are structurally and functionally a very diverse group of natural pigments of the polyene type [1]. They occur ubiquitously in all organisms capable of conducting photosynthesis, a process in which sun light is effectively converted into chemical energy. Carotenoids are important constituents of photosynthetic organelles of all higher plants, mosses, ferns and algae. They are also found in photosynthetic membranes of phototropic bacteria and cyanobacteria [2]. Although not synthesized by humans and animals, they are also present in their blood and tissues. They are important precursors of retinol (vitamin A); however, their main function in all non-photosynthetic organisms seems to be (photo)protection. Carotenoids are known to be very efficient physical and chemical quenchers of singlet oxygen (^1^O_2_), as well as potent scavengers of other reactive oxygen species (ROS) [3,4,5]. This is of special significance, because the uncontrolled generation and concomitant increase of ROS level in the body results in “oxidative stress”, an essential contributor to the pathogenic processes of many diseases. Carotenoids and some of their metabolites are suggested to play a protective role in a number of ROS-mediated disorders, such as, *i.e.*, cardiovascular diseases, several types of cancer or neurological, as well as photosensitive or eye-related disorders. However, due to numerous factors affecting the bioavailability, absorption, transport, metabolism or storage of Crts, the exact mechanisms of their functioning *in vivo* are still far from being fully understood. In the present paper, based on the data coming from epidemiological and intervention studies, as well as clinical trials, we would like to highlight the beneficial effects of Crts intake, either as supplements or as integral components of Crt-rich food, in several exemplary modern civilization diseases.

## 2. Carotenoids: Short Overview

**Figure 1:**
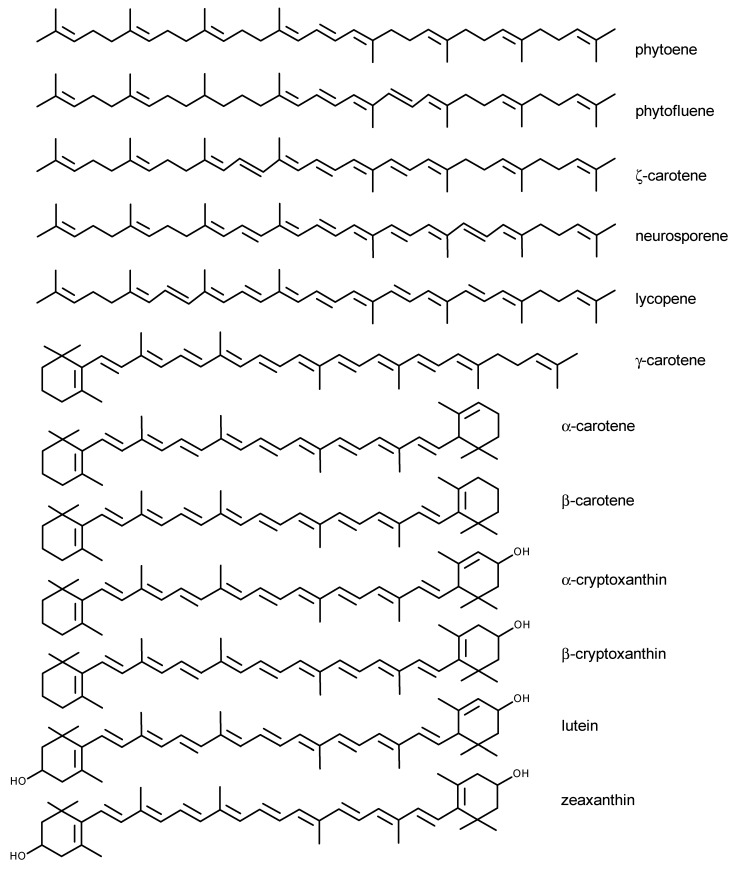
Chemical structures of major carotenoids present in human plasma.

Up to date, more than 700 Crts have been described [6], of which about 50 become constituents of the human diet [7], while only ~20 are present in human blood and tissues [8]. The most important include β-carotene, α-carotene, lycopene, lutein, zeaxanthin, β-cryptoxanthin, α-cryptoxanthin, γ-carotene, neurosporene, ζ-carotene, phytofluene and phytoene (Figure 1), all present in human plasma [7,9].

### 2.1. Chemical Structure, Function and Membrane Distribution

Most Crts exhibit a characteristic, symmetrical tetraterpene skeleton formed by the tail-to-tail linkage of the two C_20_ moieties. The linear C_40_ hydrocarbon backbone is susceptible to diverse structural modifications. These concern modifications in hydrogenation level, *cis*-*trans* isomerization, cyclization at one or both ends or the addition of side groups (often containing oxygen) with their subsequent glycosylation/acetylation. More sophisticated changes are related to the shortening or extension of the carbon skeleton resulting, in the latter case, in C_50_-Crts formation. Moreover, C_30_-Crts might be formed as products of two farnesyl units of condensation [1]. One of the most characteristic features of Crts is their strong coloration, which is a consequence of light absorption stemming from the presence of an extensive system of conjugated double bonds. The presence of such a conjugated chain is crucial for the proper functioning of Crts, which is essentially light absorption in photosynthetic organisms and (photo)protection in all living organisms. Carotenoids are also suggested to participate in: (i) the stimulation of the immune system; (ii) the modulation of intracellular signaling pathways (gap junction communication) [10]; (iii) the regulation of the cell cycle and apoptosis; (iv) the modulation of growth factors; (v) cell differentiation [11]; and (vi) the modulation of various types of receptors or adhesion molecules and many other physiologically significant processes [12].

Carotenoids, as highly lipophilic molecules, are typically located inside cell membranes. Strict hydrocarbons, such as β-carotene or lycopene, are arranged exclusively within the inner part of the lipid bilayer. “More” polar pigment molecules, containing attached oxygen atoms (e.g., lutein, zeaxanthin) are oriented roughly perpendicular to the membrane surface, exhibiting their hydrophilic parts to the aqueous environment [13,14]. Incorporation of Crts may noticeably affect the membranes’ properties (rigidity, mechanical strength, thickness, fluidity or permeability), which are crucial for their proper functioning. For example, stability and some other membrane-associated processes, such as signal transduction, are modified [15,16,17]. Subsequent changes may also result in a prominent enhancement of the membranes’ resistance toward ROS, having a positive impact on human health, since the molecular mechanism of a number of chronic diseases seems to at least partly involve ROS interactions.

### 2.2. Bioavailability and Fate in Human Body

Carotenoids are abundantly present in fresh fruits and vegetables. Yellow-orange-red fruits and green leafy vegetables are known to be especially rich in nutritional Crts. The major dietary sources of Crts have been listed recently [18,19,20]. However, as pointed out by Castenmiller and West [21] or Yeum and Russell [22], there is a number of factors that influence Crts bioavailability, absorption, breakdown, transport and storage. The type, amount and milieu in which Crts are incorporated belong to the most evident factors. Thus, Crts release from the food matrix greatly depends on their state, as well as association with other compounds (*i.e.*, proteins) [23]. The microcrystalline form of some Crts (e.g., lycopene in tomato or β-carotene in carrot) makes them less available as compared to those which are entirely immersed in lipid droplets. It is suggested that only about 5% of the Crts in whole are absorbed by the intestine, whereas >50% are from micellar solution [24]. In a number of studies, thermal treatment was shown to increase Crts accessibility, due to the disruption of cell walls and bond loosening [25,26]. Other factors, such as genetic factors and nutritional status, gender, aging or infection, also determine Crt bioavailability [21,22]. It is well-established that any disease with the abnormal absorption of fat from the digestive tract significantly affects Crts incorporation. Furthermore, interactions with drugs (e.g., sulfonamides or aspirin) were shown to decrease the availability of β-carotene [21]. Last, but not least, the interactions between different types of Crts and other food components play an important role. For example, Crts may interact with each other during absorption, metabolism and serum clearance, as was demonstrated during β-carotene and lutein administration to human subjects [27]. Another example might be the “positive” cooperation between Crts and vitamin E (see Section 4, “ROS: Antioxidants Balance”). The intestinal absorption of Crts, which are highly hydrophobic molecules, involves similar stages as in the case of dietary lipids or fat-soluble vitamins. This includes: (i) the incorporation into mixed lipid micelles in the lumen; (ii) the uptake into intestinal mucosa; (iii) the incorporation into chylomicrons; and (iv) the release into the lymph [28,29]. Following the digestion of chylomicrons by lipoprotein lipase and the release of Crts, they are further distributed mostly by the use of (very) low density lipoproteins ((V)LDL) [8,30]. Thus, the low density proteins (LDL) exhibit, among others, the highest concentration of Crts in plasma [7,24,31]. Carotenoids are mainly accumulated in the liver and adipose tissues; however, their relatively high amount was also reported for the adrenal gland, corpus luteum, testes, skin and retina (macula) in contrary to kidney and ovary, while in brain stem tissue, their concentration was below the detection limit [32,33].

## 3. Reactive Oxygen Species

### 3.1. Major Cellular Sources of ROS

Life on Earth depends on molecular oxygen, which, in all aerobic cells, is primarily utilized by specialized organelles, mitochondria, in a basic biochemical process called oxidative phosphorylation. Due to the operation in an oxygen-rich environment, the protein components of the mitochondrial electron transport chain, the elements of the citric acid cycle, together with some other enzymes (e.g., monoamine oxidase or manganese superoxide dismutase (MnSOD)) are considered as major mitochondrial sources of ROS [34,35,36,37]. The other sites of cellular ROS production include microsomes, peroxisomes or cytochrome P450. In addition, some cytosolic enzymes, for instance copper/zinc superoxide dismutase (Cu/ZnSOD), xanthin oxidase, cytochrome P450 reductase or NADPH oxidases (NOX family), have been recognized as potent ROS generators [38,39].

Apart from these, a number of exogenous physical and chemical factors, such as UV and ionizing radiation (e.g., X-rays) or chemical compounds (e.g., xenobiotics) are also known to be responsible for the stimulation of cellular ROS (or reactive nitrogen species, RNS) formation [40].

### 3.2. Types of ROS

**Figure 2:**
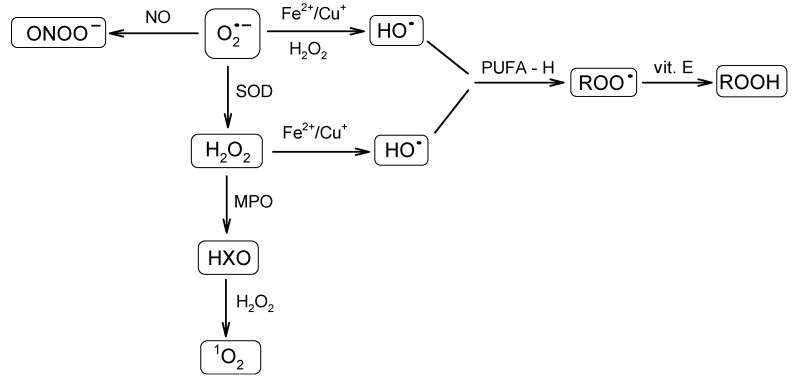
Schematic presentation of a cascade of reactions resulting in the formation of a set of reactive oxygen species (ROS) from the superoxide anion radical. SOD, superoxide dismutase; MPO, myeloperoxidase; HXO, hypohalous acid; PUFA, polyunsaturated fatty acid; ROO•, peroxyl radical; ROOH, hydroperoxide species.

A great variety of reactive oxygen-derived species are continuously generated in cells. They are capable of irreversible oxidation of fundamental biological macromolecules: proteins, lipids, nucleic acids and carbohydrates [41,42]. Radicals, such as the superoxide anion radical (O_2_^•−^) or the hydroxyl radical (HO•), and reactive non-radical species, such as hydrogen peroxide (H_2_O_2_) or singlet oxygen (^1^O_2_), are among the most abundant species. The superoxide anion radical is regarded as one of the most powerful and destructive. Because of the ubiquitous presence of the mitochondrial transport chain, it is considered as the major physiological source of O_2_^•−^. Thus, the formation of O_2_^•−^ occurs during the transportation of the electron to oxygen in the respiratory chain by previously reduced coenzymes, prosthetic groups or even xenobiotics [43]. It was found that mitochondria can generate as much as 2–3 nmol of O_2_^•−^/min per mg of protein [35]. The superoxide radical may also be generated enzymatically by respective NADPH oxidases [39,44,45] or the xanthine oxidase system. The superoxide anion radical, a relatively short-lived oxygen species [46], quickly undergoes further reactions that result in the formation of a set of other ROS (Figure 2). It may undergo dismutation to H_2_O_2_ and O_2_, either spontaneously or in the reaction catalyzed by MnSOD or Cu/ZnSOD in the mitochondrial or cytoplasmic matrix, respectively [47]. The superoxide anion radical may also react with nitric oxide (NO) to form a short-lived, but powerful, oxidant, peroxynitrite (ONOO–), capable of interactions with lipids, nucleic acids and proteins via direct oxidative reactions or radical-mediated mechanisms [48]. Furthermore, O_2_^•−^ reduces transition metals, such as iron or copper, which in the Fenton reaction together with H_2_O_2_ lead to the formation of HO•. The hydroxyl radical is famous for its extreme reactivity and harmfulness. Its lifetime was estimated to be ~2 ns in aqueous solution and its radius of diffusion ~20 Å [46,49]. Hydroxyl radical interacts with the adjacent molecules leading to their rapid oxidation. It is capable of reducing disulfide bonds and, as a consequence, protein unfolding. Its role in other deleterious processes has also been reported [50].

Another reactive species, H_2_O_2_, an example of the long-lived ROS, apart from being formed from O_2_^•−^, can also be generated directly during electron transport in mitochondria. As was estimated by Chance *et al*. [51], under physiological conditions, the amount of H_2_O_2_ generated by the mitochondrial electron transport chain corresponds to ~2% of the initial oxygen uptake. Boveris *et al*. [52] demonstrated that in rat liver, the contribution of H_2_O_2_ produced in the mitochondria, microsome, peroxisome and some enzymes to the cytosolic level of H_2_O_2_ at a pO_2_ of 158 mmHg accounts for 15%, 45%, 35% and 5%, respectively. Hydrogen peroxide is also generated as a side product during enzymatic reactions (*vide* xanthin oxidase formation). It also serves as a substrate for myeloperoxidase (MPO) in a reaction leading to hypohalous acid (HXO), which may further react with H_2_O_2_, which leads to ^1^O_2_ formation [38]. Hydrogen peroxide is regarded as a mild oxidant; however, it is able to oxidize cysteine residues in proteins [53].

Another example of non-radical ROS is ^1^O_2_. Singlet oxygen can be generated non-photochemically via an “oxidative burst”, a process in which macrophages, neutrophils or monocytes produce large amounts of ROS during phagocytosis [54,55]. Its lifetime was shown to be strongly medium-dependent and estimated in the range of 10^−6^–10^−5^ s [46,56]. Singlet oxygen predominantly interacts with double-bonded molecules (nucleic acids, polyunsaturated fatty acids (PUFA)) via energy transfer or chemical reactions [57].

Peroxyl radicals (ROO•) also constitute a very important type of ROS. They are generated during lipid auto-oxidation (usually initiated by HO•), which is an example of a chain-reaction [58]. The lifetime of ROO• was shown to be relatively long (~7 s) and the diffusion radius significant [46]. The process of lipid peroxidation might be terminated by various lipophilic antioxidants (e.g., vitamin E) [59]. Such a reaction results in the formation of hydroperoxide species (ROOH) [60], which, in turn, may rearrange to form endoperoxides and, further, metabolites, which are capable of interacting with neighboring proteins.

## 4. ROS: Antioxidants Balance

Reactive oxygen species are first and foremost described as potentially harmful agents. However, on the other hand, ROS are also known to serve positive, regulatory functions [61]. Their role in intracellular and extracellular signaling processes has already been well documented [62]. To give an example, the mitochondrial ROS generation is regarded as a component of the TNF (tumor necrosis factor)-signal transduction pathway during apoptosis [63]. Reactive oxygen species may also interfere with the expression of genes [62] or influence protein phosphorylation [53]. In some cases, ROS might be regarded as “protective” molecules having a positive impact on inflammation [64,65].

The amount of cellular ROS is kept under strict control. Each cell has a powerful defense system, a palette of diverse antioxidative molecules operating in its hydrophilic or hydrophobic environment, capable of eliminating potentially dangerous species. The type and complexity of antioxidants decide their efficiency in ROS scavenging. In some cases, they can even prevent the formation of ROS precursors. The most obvious group of antioxidants comprise enzymes, such as, for instance, those from the SOD family catalyzing conversion of O_2_^•−^ into H_2_O_2_. H_2_O_2_, depending on the place of its origin, might be decomposed either by one of the glutathione peroxidases [66] or by catalases abundant in peroxisomes or heart mitochondria. Interestingly, such catalases have not been found in the mitochondria of other tissues [67]. Another group of antioxidants include vitamins. Vitamin E (primarily α-tocopherol) is regarded as a very efficient antioxidant functioning in a hydrophobic milieu [68]. It is known to inhibit lipid peroxidation and to scavenge lipid peroxyl radicals, preventing the propagation of free radical-mediated chain reactions [59]. Furthermore, its ability to quench ^1^O_2_ or reaction with peroxynitrite have been described. As was communicated for the first time by Palozza and Krinsky [69,70], β-carotene and α-tocopherol can act synergistically as an effective “radical-trapping antioxidant” in biological membranes. The inhibition of lipid peroxidation by a combination of the two fat-soluble antioxidants was shown to be greater than the sum of the individual inhibitions. In another study [71], oxygen-containing Crt, zeaxanthin and α-tocopherol were demonstrated to deliver synergistic protection against photosensitized lipid peroxidation mediated by ^1^O_2_ and free radicals. The cooperation between hydrophilic ascorbic acid (vitamin C, reducing agent), hydrophobic α-tocopherol (vitamin E) and β-carotene (provitamin A) also led to synergistic cell protection against different RNS [72]. Furthermore, inorganic elements (e.g., selenium) and low-molecular weight organic compounds (e.g., coenzyme Q, uric acid, lipoic acid) are considered as important antioxidant molecules [73,74,75,76]. A special group of antioxidants consists of plant-derivate compounds, carotenoids and flavonoids. Carotenoids are regarded as one of the most efficient ^1^O_2_ quenchers, as well as ROS scavengers operating in cellular lipid bilayers [77]. Flavonoids, although shown to be very potent scavengers of hydroxyl and superoxide radicals, as well as active chelators of transient elements [78,79], due to their relatively poor absorption and difficulties with storage in animal tissues, are thought to have a lower contribution to the direct antioxidative protection of humans [80,81,82].

**Figure 3:**
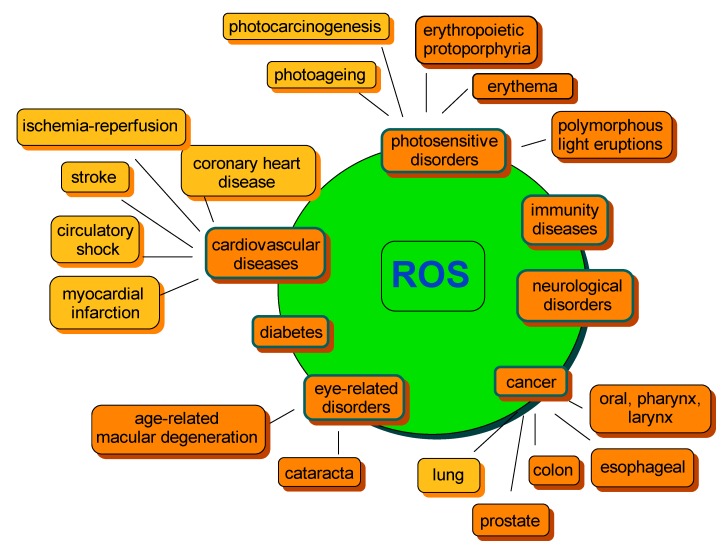
Examples of ROS-mediated disorders. The orange color indicates the beneficial effect of carotenoids on disease risk development. The yellow color indicates that an equivocal effect was reported. The diagram was constructed on the basis of information from several studies cited within the text.

A lose in balance between ROS generation and detoxification leading to ROS overproduction and, as a consequence, accumulation, may result in a range of abnormalities further associated with chronic diseases. The most common include cancer, cardiovascular diseases, photosensitivity disorders, diabetes, neurological disorders or various types of inflammation, as well as processes correlated with ageing (Figure 3) [48,82,83,84]. Therefore, compounds capable of preventing the excessive formation of ROS, thus regulating their concentration, are of special importance to human health.

## 5. Carotenoids: ROS Interactions

Carotenoids are very potent natural antioxidants. Due to their triplet energy levels lying close to that of ^1^O_2_ (1274 nm, 7849 cm^−1^ or 93.9 kJ/mole *vs*. 1380 nm, 7250 cm^−1^ or 86.7 kJ/mol for β-carotene, respectively [2,85]), they belong to the most efficient physical quenchers of ^1^O_2_, both *in vitro* and *in vivo* [4,86,87]. The process of ^1^O_2_ quenching has been shown to be very efficient, especially for Crts having 11 conjugated double bonds (≈10^10^ M^−1^·s^−1^) [77], though their protective behavior was demonstrated to be strongly medium-dependent [88,89]. In general, ^1^O_2_ deactivation is based on the conversion of an excess of energy to heat via the Crt lowest excited triplet state (^3^Crt*). The possible damaging effects of excited Crts might be ignored mostly because of their low energy and short lifetimes [2].

^1^O_2_ + Crt → ^3^O_2_ + ^3^Crt* (1)

^3^Crt* → Crt + heat (2)

Carotenoids can also act as chemical quenchers of ^1^O_2_, undergoing modifications, such as oxidation or oxygenation [3,90]. Moreover, they effectively scavenge ROS and other free radicals of different origins [4,91,92,93,94,95], delivering protection against oxidative damage to photosynthetic and non-photosynthetic organisms at all levels of complexity. There are three generally accepted major types of reactions of free radical scavenging by Crts: (i) electron transfer between the free radical (R^•^) and Crt, resulting in the formation of a Crt radical cation (Crt^•+^) (Equation 3) or Crt radical anion (Crt^•−^) (Equation 4); (ii) radical adduct formation (RCrt^•^) (Equation 5); and (iii) hydrogen atom transfer leading to a neutral Crt radical (Crt^•^) (Equation 6) [4,60].


R^•^ + Crt → R^-^ + Crt^•+^ (3)

R^•^ + Crt → R^+^ + Crt^•-^ (4)

R^•^ + Crt → RCrt^•^ (5)

R^•^ +Crt → RH + Crt^•^ (6)

The newly formed Crt radical products can undergo further transformations, leading to a variety of secondary Crt derivatives of different reactivity. This is of extreme importance, due to the fact that the newly generated Crt species may no longer act as efficient antioxidants, but turn into potentially harmful, pro-oxidant agents. For instance, one of the most studied Crt reactive species, Crt^•+^, due to its strong oxidizing properties and relatively long lifetime (ms) [96,97], is known to be able to interact with other molecules of biological importance, e.g., tyrosine or cysteine (k ~10^4^ and 10^6^ M^−1^·s^−1^, respectively) [98]. *In vivo*, the oxidation of amino acids may result in irreversible structural modifications of proteins and, thus, markedly influence their proper functioning. Moreover, the scavenging ability of Crts toward free radical species was shown to be strongly dependent on their redox properties [99], which might be modified by the presence of salts, as has been exemplified with astaxanthin [100]. In the presence of salts, its relative antioxidant ability was demonstrated to decrease as its oxidation potential decreases. The presence of salts also influenced the stability of astaxanthin radical cations and dications.

## 6. Oxidative Stress, Disease and Carotenoids

Oxidative stress is placed among the most important causes of a range of modern chronic civilization diseases. Nevertheless, there is growing evidence that antioxidants, such as Crts, may reduce or, in some cases, even prevent the development of various ROS-mediated disorders. Data coming from observational, epidemiological and intervention studies, as well as clinical trials usually support this view, although some of them are equivocal.

### 6.1. Cancer

According to the World Health Organization [101], cancer is a primary cause of death and accounted for around 13% of all deaths in 2008. It is predicted that the deaths from tumors will continue to rise to over 11 million in the year 2030. Therefore, it is so important to take adequate steps to reduce or modify the factors affecting the risk of cancer development. Undoubtedly, the simplest one seems to be the sufficient intake of fruits and vegetables rich in biologically active compounds [101]. A number of prospective studies have shown a positive correlation between the consumption of Crt-rich fruits and vegetables and a decreased risk of several types of cancer [102,103,104,105]. Thus, a large collection of data on lung cancer and dietary Crts have become available. The results usually support the observation of decreased morbidity upon β-carotene supplementation in non-smoking adults [106,107]. Furthermore, the recent case-control studies of diet and lung cancer among non-smokers confirmed an inverse correlation between lung cancer risk and intakes of food sources rich in Crts, such as α-carotene, lutein, lycopene, β-cryptoxanthin and β-carotene [108,109]. In contrast to this, the results obtained from the Alpha-Tocopherol, Beta-Carotene Cancer Prevention Trial (ATBC), involving heavy cigarette smoking men, indicated a significantly higher occurrence of lung cancer and total mortality in comparison to individuals obtaining the placebo [110]. These results were confirmed by the Beta-Carotene and Retinol Efficacy Trial (CARET) study, as well as some others, in which a combination of β-carotene and vitamin A supplementation was tested among men and women at a high risk of developing lung cancer (asbestos workers and smokers) and in subjects who consumed larger amounts of alcohol [111,112,113]. Yet, in the course of the latest detailed analyses of the results, it turns out that the unexpected “cancerogenic” (pro-oxidant) effects of Crt supplementation can be explained in terms of their strong interference with the unhealthy lifestyle of the individuals [114].

Another important collection of data comes from the studies on prostate cancer. Thus, a large number of epidemiological studies generally support the idea that several Crts, as well as Crt-rich food, could be involved in the reduction of the risk of prostate cancer [115,116]. Among various Crts, lycopene is regarded as the most potent agent against the risk of this type of tumor, in particular in its more lethal form [117]. The preclinical studies suggest several possible ways of lycopene action, indicating, at the same time, its significance in the enhancement of the oxidation stress defense system [118]. Further evidence supporting the above findings has been delivered by the recent meta-analysis of the observational studies on the role of tomato products and lycopene in the prevention of prostate cancer [119]. Furthermore, recent human intervention and clinical trials provided additional support [120,121].

The human intervention trials point to β-carotene as an important factor in the prevention of oral, pharynx and larynx cancers [122]. These data are in agreement with further observations that high consumption of fruits and vegetables results in the reduction of the risk of oral and throat cancers by about 50%. The prospective studies confirmed the results [123]. Similar data were obtained in the case of esophageal cancer. In several case-control studies, it was shown that fairly high consumption of fruits and vegetables resulted in 40%–50% lower risk of this type of cancer in comparison to low intakes [124]. Furthermore, a large body of evidence, mostly from observational studies, indicates a correlation between the intake of fruits and vegetables and the risk for colon cancer development [125,126]. Similarly, the case-control studies confirm the inverse relation between fruits/vegetable intake, serum concentration of Crts and colon cancer risk.

### 6.2. Cardiovascular and Related Disorders

Carotenoids, as highly lipophilic molecules, are expected to be particularly efficient scavengers of ROS within the hydrophobic parts of cell membranes and lipoproteins, their major transporters, reducing the possibility of the oxidation of membrane structures and the overall risk of the morbidity rate [127]. One of the classical examples of an ROS-mediated disorder is atherosclerosis (hardening of the arteries), which is the result of oxidative modification of LDL in the arterial walls leading to coronary heart disease [128]. Due to the abundance of this kind of disorder, the investigations on factors that may prevent or delay its development are of special importance. The results of the studies of the association of Crts (mainly β-carotene, α-carotene, lycopene, lutein, zeaxanthin and β-cryptoxanthin) with the risk of cardiovascular disease and atherosclerosis have been summarized by Mayne [129] and more recently by Voutilainen *et al*. [130]. On the basis of the reported data, the authors point out that there is a positive correlation between the higher intake of fruits and vegetables rich in Crts and the prevention of morbidity and mortality with relation to cardiovascular disease.

The results from intervention studies are less consistent. Thus, the results of the well-known studies conducted by the ATBC Trial, primarily designed to evaluate the effect of β-carotene on lung cancer and other types of cancer in male smokers [110], also delivered data on ischemic heart disease, stroke mortality and first major coronary events. In this case, the supplementation of 20 mg/day of β-carotene over six years resulted in an insignificant increase of ischemic heart disease and stroke mortality. As was later shown by Tornwall *et al*. [131], it also increased the post-trial risk of a first nonfatal myocardial infarction. Supplementation of a larger amount of β-carotene (50 mg/day) to adult non-smoking males and females delivered no evidence for cardiovascular disease mortality [132,133]. Furthermore, no confirmation of any relation between β-carotene intake and the five-year mortality after supplementation nor the incidence of any type of cardiovascular disease was reported by the Heart Protection Study (HPS) [134]. During the five-year studies, the male and female individuals with a previously diagnosed coronary heart disease, an occlusive arterial disease or diabetes were under close inspection. In this case, the increased risk of a fatal coronary event was reported only for male smokers with previously stated myocardial infarction [135]. The positive effects of β-carotene supplementation (additionally with or without aspirin) were also reported for individuals with ischemic heart disease. In this case, a significant reduction of a myocardial infarction risk was shown [132].

Results from the basic research, clinical and intervention studies are not consistent. They contradict each other very often. In some cases, a beneficial effect of β-carotene on cardiovascular disease has been clearly observed; in others, little or no correlation between them has been found or, even, an inverse relationship has been reported. This unexpected Crt behavior might be partially explained in terms of: (i) the generation of Crts oxygenation products of pro-oxidant activity [3]; as well as (ii) pronounced changes in their optical and chemical properties, *i.e.*, antioxidant activity. As has been demonstrated recently in the course of EPR (electron paramagnetic resonance) spin trapping experiments, the formation of H-type aggregates of Crts in aqueous media results in a considerable lowering of their antioxidant potential (e.g., lutein) or even leads to pro-oxidant behavior (*i.e*., zeaxanthin) [136]. Nevertheless, also, other Crts, such as astaxanthin, lutein or β-cryptoxanthin, rather than β-carotene, should be considered as potentially helpful agents toward cardiovascular disorders [137,138]. However, still more data need to be collected to fully explain the discrepancies between the observational and interventional data.

### 6.3. Photosensitivity Disorders

Among the physical factors noticeably affecting all kinds of biological molecules is UV radiation. The uncontrolled exposure to its natural or artificial sources may result in a range of photosensitivity disorders associated with epidermal and dermal damage. Hence, a number of degenerative changes in the cells, fibrous tissue and skin blood vessels may occur. Biologically essential UV radiation ranges from 280 up to 400 nm of the electromagnetic spectrum. The short wavelength UV-B radiation (280–315 nm) is predominately absorbed by keratinocytes in the epidermis. It may lead to sunburn (erythema), which is the first response of UV-treated skin [139]. Via direct interactions of this UV radiation with nucleic acids, it is regarded as one of the major causes of photocarcinogenesis. The longer wavelength UV-A radiation (315–400 nm) is capable of penetrating deeper parts of dermis. Apart from the undoubtedly positive effects on human health (e.g., the induction of vitamin D synthesis), it may cause the generation of ROS, which are known to be crucial in the processes of photoageing [140].

Carotenoids, due to their excellent ^1^O_2_ quenching, as well as other ROS scavenging properties, have garnered particular attention as protective agents in skin photo-related disorders. It is suggested that Crts (especially β-carotene and canthaxanthin) could act as efficient scavengers of excited triplet states of endogenous photosensitizers, such as protoporphyrin, which is accumulated in the blood and skin of patients with inherited erythropoietic protoporphyria [141]. They were also demonstrated to be effective in the treatment of polymorphous light eruptions [142].

The effect of Crts on sun erythema formation (sunburn) has been investigated intensively over the years [143,144,145,146,147,148]. In the course of a recent meta-analysis study, it has been clearly demonstrated that β-carotene supplementation does protect against sunburn in a time-dependent manner (the effected size of the protection was shown to require a minimum of 10 weeks) [149]. Human intervention studies delivered comparable results for lycopene [150,151,152]. More recently, phytoene and phytofluene, two colorless precursors of Crts, were pointed out as potentially beneficial dietary agents. Due to their spectral properties, *i.e.*, light absorption in the UV-B and UV-A range, they are expected to noticeably contribute to the photoprotective effects of Crts-rich food for skin health [148,153].

The development of skin cancer (photocarcinogenesis) is a complex process usually initiated by UV radiation [154]. The potentially advantageous effect of Crts against photocarcinogenesis remains still ambiguous. Observational studies do not confirm the role of Crts in the reduction of non-melanoma skin cancer risk [155,156,157]. On the other hand, case control studies indicate a positive correlation between basal cell carcinoma development and lutein intake [158].

The beneficial role of Crts was also postulated during the process of photoageing, which is accompanied by wrinkling, additional pigmentation, telangiectasia, dryness and skin inelasticity [159]. Nevertheless, the available experimental data are inconsistent [160]; therefore, as in the case of other light-induced skin disorders, further research to confirm the advantageous effect of Crts is required.

### 6.4. Other

The ability of Crts to act as protective agents against ROS has also been observed in eye-related disorders. In the eye lens and the macular region of the retina (yellow spot), two oxygen-containing carotenoids, lutein and zeaxanthin, are present in high concentrations. Both of them are regarded as very important components for eye health. It is suggested that their major function is protection against high-energy UV radiation that is focused onto the foveal region. Moreover, they are well-known for excellent ROS scavenging properties [77], which are of special significance, also due to the fact that biochemical processes that occur within photoreceptors (phototransduction and oxidative phosphorylation) are essential sources of ROS [161]. As has been reviewed recently [162], the data obtained from epidemiological, clinical and interventional studies demonstrate that both Crts are effective agents in reducing the risk of age-related macular degeneration, a major cause of impaired vision and blindness in the elderly, and cataracts. The role of lutein and zeaxanthin as macular pigments and their role in eye health has been summarized in detail by Loskutova *et al*. [163] and Abdel-Aal *et al*. [20].

The beneficial effects of dietary Crts have been also reported for other processes, e.g., stimulation of the immune system in inflammatory diseases or human immunodeficiency disease [164].

## 7. Conclusions

Carotenoids, being exceptionally efficient physical and chemical quenchers of ^1^O_2_ and other ROS, have garnered particular attention as potentially protective agents against ROS-mediated disorders. Up to date, in a number of epidemiological, interventional and clinical studies, a large body of data, mostly from experiments with β-carotene, lycopene, lutein and zeaxanthin, have been collected, generally supporting the observation that the adequate intake of Crt-rich fruits and vegetables or Crt supplements may significantly reduce the risk of some chronic diseases. Thus, the beneficial effects of Crt administration have been confirmed in the case of several types of cancer and cardiovascular and photosensitive disorders, as well as in eye-related diseases. Nevertheless, due to the fact that some of the results remain inconsistent, more data need to be collected before the Crt-ROS-mediated-disorder relationship will be fully recognized.
